# Effects of traditional Chinese exercise on lumbar disc herniation: A protocol of network meta-analysis of randomized controlled trials

**DOI:** 10.1097/MD.0000000000018781

**Published:** 2020-01-31

**Authors:** Sheng Yuan, Xuxin Lin, Jieshan Hong, Chen Qiu, Dong Chen

**Affiliations:** a The First Affiliated Hospital of Jinan University; b Guangzhou University of Chinese Medicine, Guangzhou, Guangdong, China.

**Keywords:** traditional Chinese exercise, lumbar disc herniation, protocol, systematic review, network meta-analysis

## Abstract

**Background:**

A large number of randomized controlled trials (RCTs) have shown that traditional Chinese exercises (TCE) have certain advantages in the treatment of lumbar disc herniation (LDH). However, due to the diversity of TCE methods, their relative effectiveness has not been studied and explained. Therefore, based on the network meta-analysis (NMA), this study will compare the differences in the effectiveness of TCE methods in the treatment of LDH, in order to provide a reference for clinical treatment.

**Methods:**

We will search PubMed, MEDLINE, Embase, the Cochrane Library, China National Knowledge Infrastructure (CHKD-CNKI), WANFANG database (Chinese Medicine Premier), Chinese Biomedical Literature database VIP for relevant RCTs of ACU treatments for POP, from their inceptions to March 18, 2019. STATA 15.0 and GEMTC software will be used to perform a NMA. The evidence will be evaluated by the Grading of Recommendations Assessment, Development, and Evaluation approach and the type 1 error rate will be assessed by trial sequential analysis.

**Results:**

The results of this review will be submitted to a recognized journal for publication.

**Conclusion:**

This proposed systematic review will evaluate the different advantages of various types of TCE in the treatment of LDH.

## 1 Introduction

Lumbar disc herniation (LDH), a common cause of low back pain, refers to the disruption of the normal structure of the intervertebral disc and causes the lumbar disc to protrude or the nucleus of the medulla, which may exert pressure on the spinal cord or nerve root and cause radiating pain.^[1]^ As a common disease, LDH affects 2% to 3% of the world's population, while the incidence of LDH in China is approximately 7.62%.^[2,3]^ LDH is a common and frequently occurring disease with a person's lifetime prevalence of 60% to 90% that has brought economic and psychologic burdens to many families and a heavy burden on social care.^[4,5]^

At present, the clinical treatment of LDH is mainly performed by percutaneous, endoscopic, minimally invasive surgery, discectomy, and other surgical treatments. Study showed that only 10% to 20% of patients with LDH clinically required surgery, but surgical treatments have some disadvantages, such as discectomy may damage the paravertebral muscles, and may lead to lumbar instability after surgery, which results in poor effectiveness and even recurrence in more patients after surgery.^[6,7]^ Most patients could reduce or alleviate the symptoms of pain through analgesia, manual therapy, and acupuncture treatment.^[8]^ Traditional Chinese exercise (TCE), with a long history, it is a combination of movement and static, rigid, and flexible movement mode, represented by Tai Chi, Baduanjin, Wu Qin Xi, Yi Jin Jing, and so on. As a simple and cost-effective treatment, TCE could relax muscles and tendons, adjust spine balance to prevent, and treat lumbar disc disease,^[8]^ and relevant clinical studies have proven its effectiveness.^[9,10]^ Long-term Taijiquan exercise will relax muscles, improve lumbar muscle strain, and relieve waist pain.^[11]^ High-intensity Taijiquan training could change the hemodynamics of elderly people with transient weakness to improve physical function.^[12]^ Related research shows that Baduanjin could improve bone mineral density, improve balance, and relieve chronic low back pain.^[13]^ In addition, regular exercise of Baduanjin could improve the proprioception of low limbs, enhance cardiopulmonary endurance, flexibility, and low limb explosiveness for college students.^[14]^

In recent years, a large number of randomized controlled trials and meta-analyses have shown that TCE have certain advantages in the treatment of LDH.^[15]^ However, due to the diversity of TCE methods, its relative effectiveness have not yet been studied and explained. Therefore, based on the network meta-analysis (NMA) analysis method, this study will compare the differences in the effectiveness of TCE.

## 2 Methods

### 2.1 Study registration

The protocol has been registered on PROSPERO CRD42019129681 (https://www.crd.york.ac.uk/PROSPERO/).

### 2.2 Eligibility criteria

#### 2.2.1 Participants

All patients are adults with LDH (as diagnosed using any recognized diagnostic criteria). Patients with LDH are associated with severe underlying diseases or complications, such as acute cardiovascular and cerebrovascular diseases, severe hepatic and renal insufficiency, diabetes, severe endocrine diseases, tumors, severe infections, or septic shock will be excluded.

#### 2.2.2 Interventions

The experimental group will be divided into 4 groups, treated with TCE, including Wuqinxi, Baduanjin, Yijinjing, Tai Chi, and so on. The control group is treated with non-TCE or pharmacotherapies. Pharmacotherapies include drugs recommended in international or domestic authorized clinical guidelines. Studies which combine TCE with pharmacotherapy are required to use the same pharmacotherapy in both the experimental and the control groups.

#### 2.2.3 Outcomes

The primary outcomes are total effective rate, visual analog score, Japanese Orthopaedic Association scores and Oswestry disability index. The secondary outcome is electromyogram scores and adverse reactions.

### 2.3 Search strategy

We will search the following electronic bibliographic databases: PubMed, MEDLINE, Embase, the Cochrane Library, China National Knowledge Infrastructure (CHKD-CNKI), Wanfang database (Chinese Medicine Premier), Chinese Biomedical Literature database, and VIP. All of them must have been published in English or Chinese, and will be searched from inception to March 18, 2019.

### 2.4 Study selection and data extraction

Two reviewers will independently conduct literature screening, data extraction and check. In case of disagreements, a third reviewer will participate in consensus conferences. In literature screening, we should read the title and abstract of the retrieved research first, exclude the irrelevant literature, and then read the full text of the included research to determine whether it is included. The following information will be extracted from the included trials using a PRISMA flowchart and Excel 2010: author, age, the participants’ characteristics, methods, duration of symptoms, intervention procedures, and outcomes.

### 2.5 Risk of bias assessment

Describe the method of assessing risk of bias or quality assessment. State which characteristics of the studies will be assessed and any formal risk of bias tools that will be used. The methodologic quality of all the random control trials will be assessed based on the Cochrane Handbook 5.1.0 for Systematic Reviews of Interventions, which comprises 7 items: random sequence generation, allocation concealment, blinding of participants and personnel, blinding of outcome assessment, incomplete outcome data, selective reporting and other sources of bias. The studies will be evaluated as being of “low risk of bias,” “high risk of bias,” or “unclear risk of bias.”^[16]^ At the same time, the included clinical trials will be awarded a score from 0 to 5 points according to Jadad scale evaluation criteria, which include reference to the generation of random sequences, blind enforcement, and withdrawal.^[17]^

### 2.6 Statistical analysis

#### 2.6.1 Pairwise meta-analysis

We will perform pairwise meta-analyses of direct evidence using the random-effects model with STATA 15.0 software (STATA Corp, College Station, TX), while a random effects NMA within a Bayesian framework will be performed. Where different measures are used to assess the same outcome, dichotomous outcomes data will be analyzed by calculating Mantel–Haenszel odds ratios and continuous outcomes will be pooled with standardized mean difference. We will present 95% confidence intervals (CIs) for all outcomes. The data will be managed on an intention to treat basis as far as possible. Attempts will be made to obtain missing data from the trial authors. Where data are unobtainable, we will assume that an event, without a reported outcome, has not occurred in participants and we will analyze only the available data.

#### 2.6.2 Network meta-analysis

The STATA 15.0 and GEMTC software (Generate Mixed Treatment Comparisons, http://drugis.org/gemtc-gui) will be used to perform NMA to compare direct and indirect evidence. After comparing multiple interventions, we will calculate the surface under the cumulative ranking curve (SUCRA), the SUCRA values will be used to rank the pros and cons of interventions. Trial sequential analysis (TSA) 0.9 (Copenhagen Trial Unit, Copenhagen, Denmark) will be used to perform TSA for assessing the type 1 error rate for selected pooled estimates.^[18]^

#### 2.6.3 Measures for inconsistency

For a closed loop of 3 treatments, the inconsistency between direct and indirect evidence will be directly examined. For the closed loop formed by the 4 studies, it can be divided into 2 closed triangular loops to test the inconsistency between direct evidence and indirect evidence. In every closed loop, we will use “inconsistency factors” (IF) value to calculate the absolute difference, when the IF value is close to 0 and its 95% CI crosses 0, this indicates that the degree of inconsistency is low, and the consistency model will be selected. On the contrary, when the IF value is far from 0 and its 95% CI does not contain 0, the degree of inconsistency is larger, and the inconsistency model will be selected.

#### 2.6.4 Quality of evidence

The quality of evidence will be assessed with the Grading of Recommendations Assessment, Development, and Evaluation (GRADE) approach.^[19]^

## 3 Discussion

Systematic evaluation showed that TCE therapy was effective and safe for LDH treatment, but there is no direct comparison between different TCE methods, so clinicians could not judge the therapeutic value of different forms of TCE therapy, which is not conducive to the selection of the best exercise therapy in Chinese medicine. Therefore, NMA will be used to compare the effectiveness differences among TCE methods, thus providing reliable evidence-based medical evidence for clinical promotion and evaluation of the effectiveness of TCE on LDH. The agreement has been registered with PROSPERO, the internationally anticipated system review registry, and will be implemented strictly in accordance with NMA procedures. In addition, the GRADE method will be used to assess the quality of evidence for key results. NMA will build on the PRISMA extension statement and will be used for reporting systematic evaluations in conjunction with a NMA of medical interventions. However, this study will only include Chinese and English literature, which will lead to selection bias. In addition, most of the included literature has different treatment times for TCE therapy, which may have an impact on the effectiveness. Therefore, we will use subgroup analysis to reduce inconsistencies. In conclusion, we hope that this study will provide more evidence for the treatment of LDH with TCE.

## Author contributions

**Data curation:** Chen Qiu.

**Formal analysis:** Sheng Yuan.

**Methodology:** Sheng Yuan, Xuxin Lin.

**Funding acquisition:** Dong Chen.

**Project administration:** Dong Chen.

**Validation:** Dong Chen.

**Writing – original draft:** Sheng Yuan, Xuxin Lin.

**Writing – review & editing:** Sheng Yuan, Jieshan Hong.
